# Factors associated with choosing diagnostic radiology as a future specialty among Palestinian medical students: a multicenter cross-sectional study

**DOI:** 10.1371/journal.pone.0361927

**Published:** 2026-10-29

**Authors:** Ibrahim Ammar AbuAttallah, Jana A. Qrareya, Reem S. Adawi, Jehad Khamaysa, Mahdi Thabet Aljamal

**Affiliations:** 1 Faculty of Medicine, Arab American University, Jenin, Palestine; 2 Radiology Department, Turki Governmental Hospital, Tubas, Palestine; University of Pisa, ITALY

## Abstract

**Background:**

Understanding factors influencing specialty choice is essential for workforce planning and curriculum development. Recruitment into diagnostic radiology remains challenging in several regions, particularly in low- and middle-income settings. This study aimed to identify factors associated with Palestinian medical students’ consideration of diagnostic radiology as a future specialty, with particular emphasis on educational exposure, perceptions of artificial intelligence (AI), and cognitive influences.

**Methods:**

A multi-institutional cross-sectional study was conducted to evaluate factors influencing the choice of diagnostic radiology as a future specialty among Palestinian medical students between 2 November 2025 and 5 February 2026. Data were collected using a previously validated questionnaire adapted from Almuhanna et al. Descriptive statistics were performed, followed by bivariate analyses and multivariable logistic regression to identify independent predictors of considering radiology as a future specialty. Adjusted odds ratios (OR) with 95% confidence intervals (CI) were reported.

**Results:**

A total of 427 students participated (56.7% female; mean age 21.75 ± 2.1 years). Among 300 valid responses regarding radiology intention, 102 students (34.0%) reported considering radiology as a future specialty. In multivariable analysis, lack of exposure to radiology research (OR = 0.14, 95% CI 0.06–0.34, p < 0.001) and lack of preclinical radiology teaching (OR = 0.26, 95% CI 0.12–0.54, p < 0.001) were independently associated with a lower likelihood of considering radiology. Enjoyment of diagnostic problem-solving tasks (OR = 3.35, 95% CI 1.39–8.09, p = 0.007) and exposure to tele-radiology (OR = 3.96, 95% CI 1.72–9.12, p = 0.001) were independently associated with higher likelihood. Lifestyle, impact on patient care, and work environment were the most frequently reported motivating factors.

**Conclusions:**

Palestinian medical students’ interest in radiology is strongly associated with modifiable educational exposures, cognitive engagement in diagnostic reasoning, and perceptions of evolving technological practice. These findings support the potential value of structured undergraduate radiology exposure, research opportunities, and mentorship initiatives to improve awareness and interest in radiology.

## Introduction

Choosing a medical specialty is one of the most important career decisions made by medical students and is influenced by multiple personal, educational, and healthcare system factors. Although specialty choice is an individual decision, the collective distribution of career choices has important implications for healthcare workforce planning [1].

Concerning choosing radiology as a specialty, even the level of income [2], personal interests [3], and factors related to a medical student’s character may play a role [4,5]. During medical school, these students build up multiple thoughts regarding each specialty, some of which may be correct, and others may not. Of these, for example, are the lifestyle in the future, work-life balance, and fewer working hours compared with different specialties [6]. According to Alsubai et al., sex also influences specialty choice [7].

Other important considerations include the growing demand for radiologists within modern healthcare systems, as radiology is a critical tool for diagnosis and management in different diseases and different fields, placing radiology at the center of modern medical practice. Despite this increasing demand, recruitment into radiology remains insufficient in many countries, highlighting the need to better understand students’ career preferences. [8].

Still, there are multiple obstacles for medical students to choose radiology, either personal or related to the lack of opportunities to pursue the specialty. This underscores the necessity of focused research into the variables that influence students’ interest in this area [1]. Many studies questioned this and found that intellectual and technical challenges, a positive work environment, and a better lifestyle all played encouraging factors for medical students to choose radiology as a specialty. In contrast, less patient contact and poor radiology rotation in medical school were found to discourage medical students from choosing radiology as a specialty [9,10].

The future of radiology is affected by technology and artificial intelligence. Artificial intelligence applications in radiology are more than image interpretation; they also support clinical decisions, help in reporting, and structure workflow, emphasizing how radiology is a field that is continuously changing and guided by technology [11].

A recent cross-sectional study in Saudi Arabia by Almuhanna et al. (2025) found that the main factors influencing medical students’ interest in diagnostic radiology were work environment, intellectual challenge, lifestyle factors, and work– life balance. In contrast, less contact with patients and medical students’ worries about radiation exposure were discouraging. The authors of this study point to the lack of institutional and regional research on this subject, underscoring the importance of further study in different geographic and cultural contexts [12].

Similarly, a study conducted in 2020 reported that a lack of awareness and misconceptions about radiation exposure were significant barriers to pursuing diagnostic radiology; greater exposure and awareness, particularly regarding interventional radiology, were associated with increased interest in the field. The study further emphasized the need for region-specific research in comparable healthcare and educational contexts [13].

In Palestine, research studying factors influencing medical students’ decisions to choose radiology as a specialty is still limited. Specialty choices of Palestinian medical students may be influenced by multiple factors, for example, clinical exposure opportunities, availability of imaging technologies, and recognition of radiology’s importance in healthcare. Therefore, this study aimed to identify factors associated with considering diagnostic radiology as a future specialty among Palestinian medical students, with particular emphasis on educational exposure, perceptions of artificial intelligence, tele-radiology, and cognitive influences.

## Methods

### Study design and settings

A multicenter cross-sectional study was conducted from 2 November 2025 to 5 February 2026 to assess factors associated with considering diagnostic radiology as a future specialty among Palestinian medical students and medical interns. The study was conducted using an online questionnaire and recruited medical students from the second through sixth academic years and medical interns from all accredited medical schools in Palestine (West Bank and Gaza Strip). The questionnaire link was disseminated through official and student-led WhatsApp groups, Telegram groups, Facebook pages, and student representatives.

### Study population

The target population included undergraduate medical students in their second through sixth academic years from all Palestinian medical faculties and medical interns who graduated from Palestinian medical schools. All eligible medical students in medical college, irrespective of their university affiliation, were able to participate. They were required to give voluntary informed consent. First-year students, students from non-medical or other health-related faculties, those studying medicine outside Palestine, and individuals who declined consent or submitted incomplete questionnaires were excluded. As such, this approach aimed to improve the quality of data and the broad representation of academic levels.

### Sample size and sampling techniques

A minimum of 384 participants was the required sample size, which was calculated using OpenEpi utilizing a 95% confidence level, 5% margin of error, and an estimated proportion of 0.5. To account for potential non-responders, 12% were added; our target was 431 medical students and interns. A non-probability convenience sampling approach was used, distributing the questionnaire via WhatsApp, Facebook, and Telegram, whereby the questionnaire was distributed to medical students across all academic years. Representation from different academic levels was achieved through the broad dissemination of the survey link, without pre-specified quotas for each year.

### Study tool, validity, and reliability

Data were collected using a self-administered online questionnaire adapted from the instrument used by Almuhanna et al. (2025) [12], which assessed factors influencing medical students’ choice of diagnostic radiology as a specialty at Imam Abdulrahman bin Faisal University, Saudi Arabia. The original questionnaire covered specialty interests, exposure to radiology, perceptions of radiologists’ roles, factors influencing radiology career preferences, and attitudes toward artificial intelligence and technological developments.

The original questionnaire was supplemented with two additional items exploring cognitive and visual–spatial factors relevant to interest in diagnostic radiology. These items were developed collaboratively by the research team following a focused literature review and reviewed by two consultant radiologists for relevance and clarity. Minor wording revisions were made based on their feedback before the questionnaire was finalized.

The questionnaire was administered in English. No additional pilot testing or psychometric validation of the adapted questionnaire was performed. The added items were exploratory, and the absence of further validation was considered a limitation when interpreting the findings. The questionnaire as administered is provided in S1 Appendix.

### Study variables

There were many variables in the study to assess the influencing factors on medical students’ intention to choose diagnostic radiology as a specialty. Demographics, academic level, exposure to radiology, perceptions of radiology practice, perceived impact of artificial intelligence, and cognitive and personal factors affecting career choice were independent variables. The intention to select diagnostic radiology as a future medical specialty was the primary variable measured using a binary (yes/no) response, as defined in the questionnaire. The authors extended upon the initial questionnaire, including themes emerging beyond the original instrument, by devising additional items focused on cognitive and personal influences, guided by current literature and the evolving role of cognitive processes in radiology decision-making.

The final questionnaire covered the following domains

- Demographic characteristics
- Current specialty interests
- Exposure to radiology
- Medical students’ perception and awareness of the radiologists’ role
- Factors influencing preference for diagnostic radiology
- Artificial intelligence, technology, and the future of radiology
- Cognitive and personal influences on interest in radiology

Operational definitions and standardized measures were applied across variables to ensure consistent data collection and interpretation, thereby enhancing the transparency and reproducibility of the study.

### Statistical analysis

Survey responses were exported from the online survey platform and analyzed using IBM SPSS Statistics, version 27.0 (IBM Corp., Armonk, NY, USA), under an institutional license held by Arab American University, Palestine. Descriptive statistics were used to summarize participant characteristics and study variables using frequencies and percentages. Bivariate associations between categorical variables and considering radiology as a future specialty were assessed using Pearson’s chi-square test or Fisher’s exact test, as appropriate. Variables with a p-value ≤ 0.20 in bivariate analysis, along with clinically relevant factors, were entered into a multivariable binary logistic regression model to identify independent predictors of considering radiology as a future specialty. Odds ratios with 95% confidence intervals were reported. Model fit was assessed using the Omnibus test, Hosmer–Lemeshow test, and pseudo-R^2^ statistics. A p-value < 0.05 was considered statistically significant. Missing data were handled using a complete-case analysis approach. Participants with incomplete responses for variables included in the multivariable logistic regression model were excluded from the adjusted analysis. No imputation techniques were applied, as the proportion of missing data was deemed acceptable for exploratory analysis.

### Ethical approval and informed consent

The study was conducted in accordance with the Declaration of Helsinki. Ethical approval was obtained from the Institutional Review Board of Arab American University (approval number J-2025/A/67/N). Electronic informed consent was obtained from all participants before they completed the online questionnaire. Participation was voluntary, and confidentiality and anonymity were maintained.

## Results

### Descriptive data analysis

#### Participant recruitment.

Participant recruitment and inclusion are summarized in Fig 1. A total of 436 medical students accessed the survey, of whom 427 completed the questionnaire. Of these, 300 participants provided valid responses regarding their intention to pursue diagnostic radiology and were included in the primary analysis. Complete data for all covariates were available for 267 participants, who were included in the multivariable logistic regression analysis.

**Fig 1:**
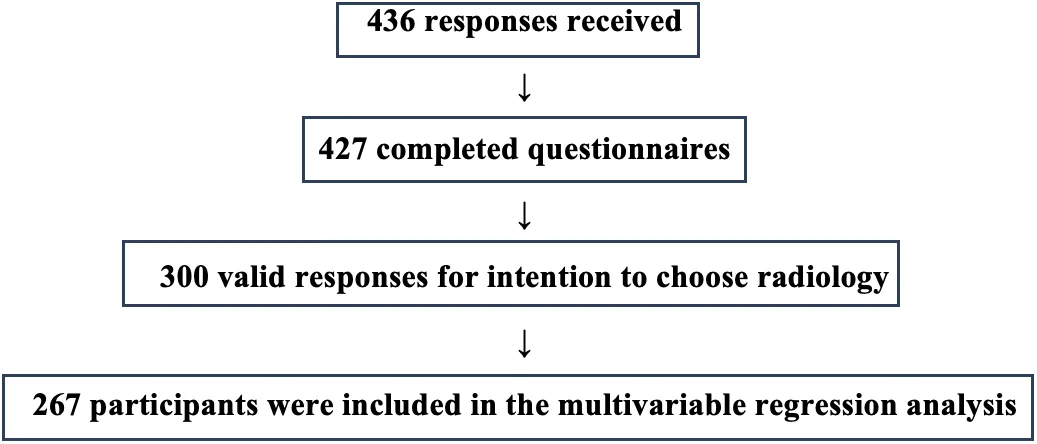
Flow diagram of participant recruitment, inclusion, and final analytical sample in the cross-sectional study.

#### Participant characteristics.

The mean age of participants was 21.75 ± 2.1 years (range, 18–30 years). Most participants were female (242/427, 56.7%) and single (416/427, 97.4%). The distribution across academic years was relatively balanced, with students from the second through sixth years and interns represented. Detailed demographic characteristics are presented in Table 1.

**Table 1:** Demographic and academic characteristics of participants.

Variable	Category	n	%
Gender	Male	185	43.3
Gender	Female	242	56.7
Marital status	Single	416	97.4
Marital status	Married	10	2.3
Marital status	Divorced	1	0.2
Monthly income (NIS)	< 2,500	228	53.4
Monthly income (NIS)	2,500–5,000	114	26.7
Monthly income (NIS)	> 5,000	85	19.9
Primary source of income	Family support	391	91.6
Primary source of income	Scholarship	18	4.2
Primary source of income	Part-time work	17	4.0
Year of study	2nd year	64	15.0
Year of study	3rd year	89	20.8
Year of study	4th year	73	17.1
Year of study	5th year	79	18.5
Year of study	6th year	92	21.5
Year of study	Intern	30	7.0
University	Arab American University (AAUP)	217	50.8
University	An-Najah National University (NNU)	105	24.6
University	Al-Quds University	34	8.0
University	Palestine Polytechnic University	24	5.6
University	Hebron University	14	3.3
University	Washington University	9	2.1
University	Gaza universities	24	5.6
Grade Point Average (GPA)	Acceptable	6	1.5
Grade Point Average (GPA)	Good	144	37.1
Grade Point Average (GPA)	Very good	238	61.3

N = 427. GPA percentages use n = 388. NIS, Israeli new shekel; GPA, grade point average.

#### University distribution.

Participants were recruited from all accredited Palestinian medical schools. The largest proportion was from the Arab American University (217/427, 50.8%), followed by An-Najah National University (105/427, 24.6%), while the remaining participants were enrolled at other Palestinian medical schools (Table 1).

#### Socioeconomic characteristics.

More than half of the participants (228/427, 53.4%) reported a monthly family income of less than 2,500 NIS, whereas 85 (19.9%) reported a monthly income exceeding 5,000 NIS. Family support was the primary source of income for most participants (391/427, 91.6%) (Table 1).

### Current specialty interest

Of the 247 (57.8%) students interested in a particular specialty at this stage, 138 (32.3%) were interested in medicine, 139 (32.6%) in surgery, and 150 (35.1%) in both specialties. The most preferred specialties were internal medicine (12.4%), general surgery (11.2%), cardiac surgery (11.0%), dermatology (7.3%), and neurosurgery (6.7%). The participants’ interest has no significant association with choosing radiology as a future specialty. (Fig 2)

**Fig 2:**
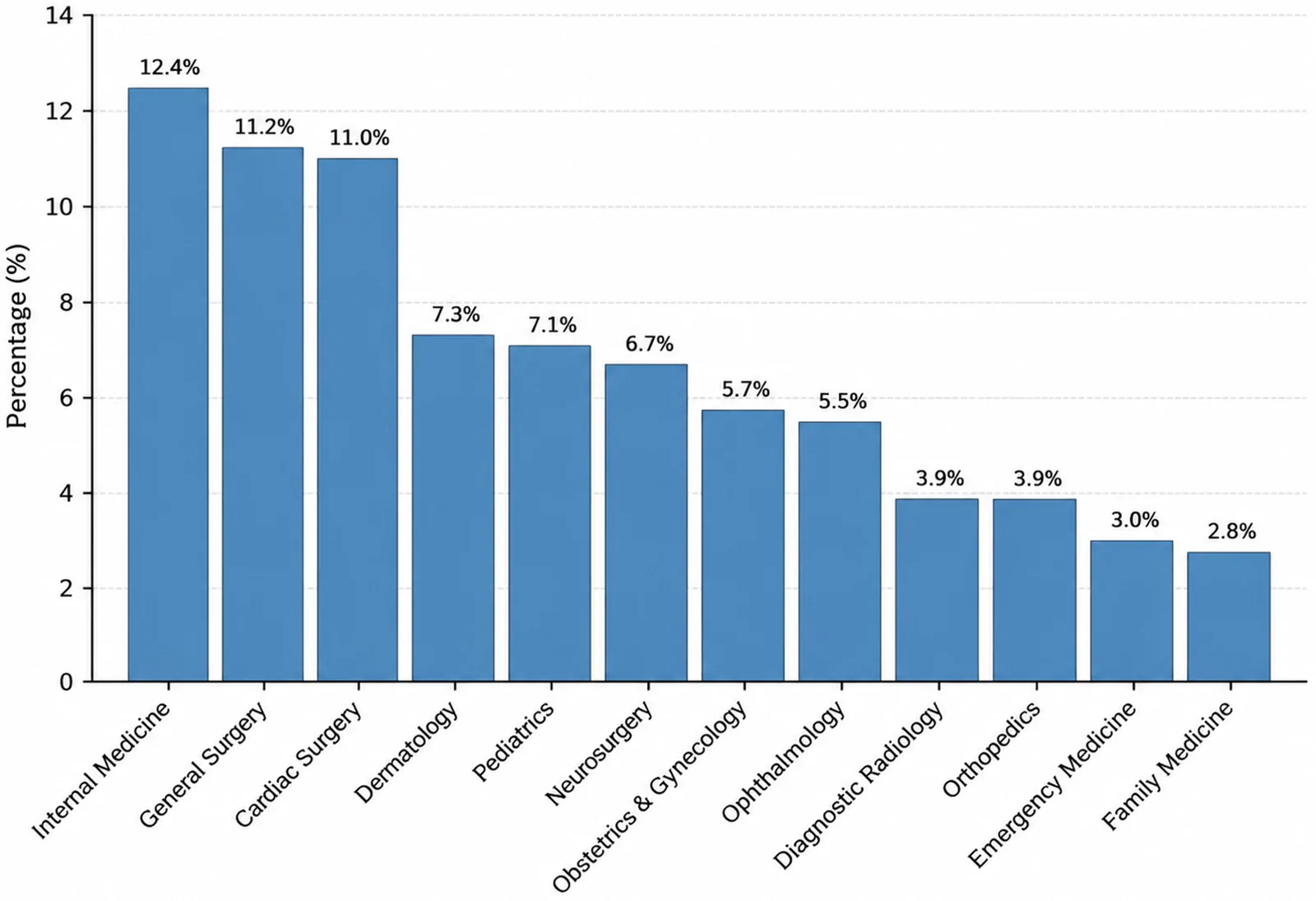
Distribution of preferred medical specialties among participating students.

Among the 300 valid responses, only 102 participants (34.0%) reported the intention of pursuing radiology as a future specialty, whereas 198 participants (66.0%) did not have such an intention. The proportion of students considering diagnostic radiology as a future specialty is presented in Figure 3.

**Fig 3:**
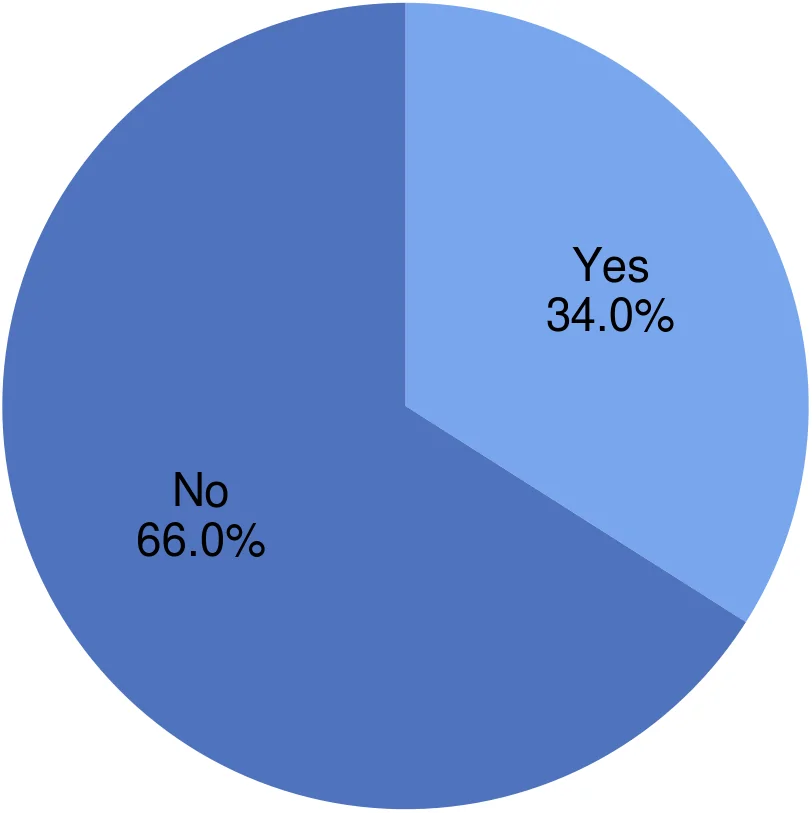
The proportion of students considering diagnostic radiology as a future specialty.

Regarding the factors influencing medical students’ choice of specialty, 67.7% chose work/life balance, followed by income potential or future earnings (66.5%), (65.8%) diversity within the field (65.1%), working environment, and relationships. Current exposure/knowledge of the field (59.3%), (58.5%) status of the specialty, and interest in acute patient management (57.4%). Table 2

**Table 2:** Reported influences on general medical specialty choice.

Factor influencing specialty choice	Reported percentage
Work–life balance	67.7
Income potential/future earning	66.5
Diversity within the field	65.8
Working environment and relationships	65.1
Current exposure/knowledge of the field	59.3
Status of the specialty	58.5
Interest in acute patient management	57.4
Extent of patient contact	56.7
Length of training	54.8
Competition for training scheme	51.8
Academic examination results	47.1
Mentor/role-model influence	46.6
Potential litigation/legal issues	40.0
Family expectations	36.8

### Association between considering radiology as a future specialty and categorical variables

Students’ intention to choose radiology as a future specialty was significantly associated with multiple factors. Greater knowledge of patient management (*p* = 0.04), current exposure to or knowledge of the radiology field (*p* = 0.011), and perceived work–life balance (*p* < 0.001) were associated with an increased likelihood of considering radiology. In contrast, stronger family expectations (*p* = 0.008), mentor or role-model influence (*p* = 0.01), and concerns regarding potential litigation and legal issues (*p* = 0.01) were associated with a decreased likelihood of choosing radiology Table 3.

**Table 3:** Associations between general specialty choice factors and consideration of radiology.

Factor	Influence level	Not considering radiology(n = 198), n (%)	Considering radiology(n = 102), n (%)	p-value reported in manuscript
Extent of patient contact	No influence	39 (19.7)	12 (11.8)	0.040
Extent of patient contact	Minor influence	51 (25.8)	36 (35.3)	
Extent of patient contact	Moderate influence	80 (40.4)	32 (31.4)	
Extent of patient contact	Strong influence	28 (14.1)	22 (21.6)	
Family expectations	No influence	70 (35.4)	19 (18.6)	0.008
Family expectations	Minor influence	55 (27.8)	45 (44.1)	
Family expectations	Moderate influence	53 (26.8)	26 (25.5)	
Family expectations	Strong influence	20 (10.1)	12 (11.8)	
Current exposure/knowledge	No influence	29 (14.6)	8 (7.8)	0.011
Current exposure/knowledge	Minor influence	44 (22.2)	40 (39.2)	
Current exposure/knowledge	Moderate influence	86 (43.4)	34 (33.3)	
Current exposure/knowledge	Strong influence	39 (19.7)	20 (19.6)	
Work–life balance	No influence	19 (9.6)	3 (2.9)	< 0.001
Work–life balance	Minor influence	42 (21.2)	44 (43.1)	
Work–life balance	Moderate influence	77 (38.9)	26 (25.5)	
Work–life balance	Strong influence	60 (30.3)	29 (28.4)	
Mentor/role-model influence	No influence	48 (24.2)	14 (13.7)	0.010
Mentor/role-model influence	Minor influence	55 (27.8)	46 (45.1)	
Mentor/role-model influence	Moderate influence	66 (33.3)	33 (32.4)	
Mentor/role-model influence	Strong influence	29 (14.6)	9 (8.8)	
Potential litigation/legal issues	No influence	58 (29.3)	17 (16.7)	0.010
Potential litigation/legal issues	Minor influence	60 (30.3)	46 (45.1)	
Potential litigation/legal issues	Moderate influence	58 (29.3)	26 (25.5)	
Potential litigation/legal issues	Strong influence	22 (11.1)	13 (12.7)	

### Exposure to radiology

Among participants with valid responses, the majority reported some exposure to radiology (300 of 427, 70.3%). However, formal or structured exposure was limited. The nature of exposure was mostly through preclinical radiology lectures (68.4%). Only 11.2% reported involvement in radiology-related research, 9.4% had attended radiology conferences, and 8.0% participated in radiology-related group events. The lowest level of exposure was observed for clinical radiology rotations, with only 4.4% of students reporting completion of a clinical rotation, while 23.4% reported undertaking a radiology elective Table 4.

**Table 4:** Radiology exposure and reported educational experiences.

Variable	Category	n	%
To date, have you had any exposure to radiology?	Yes	300	70.3
To date, have you had any exposure to radiology?	No	127	29.7
Have you attended pre-clinical radiology lectures?	Yes	292	68.4
Have you attended pre-clinical radiology lectures?	No	135	31.6
Have you participated in radiology-related research?	Yes	48	11.2
Have you participated in radiology-related research?	No	379	88.8
Have you attended radiology conferences?	Yes	40	9.4
Have you attended radiology conferences?	No	387	90.6
Have you participated in radiology group events?	Yes	34	8.0
Have you participated in radiology group events?	No	393	92.0
Have you completed a clinical rotation in radiology?	Yes	19	4.4
Have you completed a clinical rotation in radiology?	No	408	95.6
Have you taken an elective rotation in radiology?	Yes	100	23.4
Have you taken an elective rotation in radiology?	No	327	76.6
Why did you engage in radiology-related experience?	No prior experience	147	34.4
Why did you engage in radiology-related experience?	Considering radiology as a specialty choice	51	11.9
Why did you engage in radiology-related experience?	To expand exposure to radiology, particularly clinical exposure	52	12.2
Why did you engage in radiology-related experience?	Not choosing radiology as a specialty, but recognizing its importance	177	41.5
Did your experience influence your career decision?	Toward radiology	81	52.3
Did your experience influence your career decision?	Away from radiology	72	46.5
Perceived adequacy of exposure to radiology	Too little	130	44.2
Perceived adequacy of exposure to radiology	Just right	144	49.0
Perceived adequacy of exposure to radiology	Too much	20	6.8

### Association between considering radiology as a future specialty and exposure to radiology modalities

Exposure-related factors were significantly associated with students’ intention to choose radiology as a future specialty. Students who reported **any exposure to radiology**, attendance at **preclinical radiology lectures**, participation in **radiology-related research**, and completion of a **radiology elective** were significantly more likely to consider radiology as a future career compared with those without such exposure. (*p* = 0.003, 0.003, < 0.01, < 0.01) Table 5.

**Table 5:** Associations between radiology exposure and consideration of radiology.

Variable	Category	Not considering radiology(n = 198), n (%)	Considering radiology(n = 102), n (%)	p-value
Any exposure to radiology	No	69 (34.8)	19 (18.6)	0.003
Any exposure to radiology	Yes	129 (65.2)	83 (81.4)	
Pre-clinical radiology lectures	No	74 (37.4)	21 (20.6)	0.003
Pre-clinical radiology lectures	Yes	124 (62.6)	81 (79.4)	
Radiology-related research	No	185 (93.4)	76 (74.5)	< 0.001
Radiology-related research	Yes	13 (6.6)	26 (25.5)	
Radiology elective	No	160 (80.8)	58 (56.9)	< 0.001
Radiology elective	Yes	38 (19.2)	44 (43.1)	

### Perceptions and awareness of radiologists’ role

The majority of students agreed that radiologists play an important role in diagnosis and management (92.5%). Awareness of the distinction between a radiologist and a radiographer was reported by 62.5% of participants. The majority (85.2%) identified radiologists as responsible for diagnosing patients (85.2%) and directing radiographers in optimizing imaging parameters (77.5%). A large proportion also recognized radiologists’ responsibility for radiation protection during X-ray or CT examinations (74.0%) and their role in performing conventional radiographic examinations (71.9%). Approximately half of the respondents reported that radiologists meet with patients (52.9%) and perform surgical or interventional procedures (44.7%), while fewer perceived radiologists as providing direct medical treatment (37.2%) or direct surgical treatment (41.7%) Table 6.

**Table 6:** Participant perceptions of radiologist roles.

Variable	Category	n	%
Radiologists play an important role in diagnosis and management	Agree	395	92.5
Radiologists play an important role in diagnosis and management	Disagree	17	4.0
Radiologists play an important role in diagnosis and management	Neither agree nor disagree	15	3.5
Awareness of the difference between a radiologist and a radiographer	Yes	267	62.5
Awareness of the difference between a radiologist and a radiographer	No	74	17.3
Awareness of the difference between a radiologist and a radiographer	Not sure	86	20.1
Reported radiologist role	Diagnose patients	364	85.2
Reported radiologist role	Direct medical treatment	159	37.2
Reported radiologist role	Direct surgical treatment	178	41.7
Reported radiologist role	Meet with patients	226	52.9
Reported radiologist role	Perform surgical procedures	191	44.7
Reported radiologist role	Perform conventional X-ray/radiographic examinations	307	71.9
Reported radiologist role	Direct radiographers in optimizing imaging parameters	331	77.5
Reported radiologist role	Responsible for radiation protection during X-ray or CT examinations	316	74.0

### Association between considering radiology as a future specialty and perceptions of the radiologist’s role

Students’ perceptions of the radiologist’s role were associated with their intention to consider radiology as a future specialty. Awareness of the distinction between a radiologist and a radiographer was more common among students considering radiology (70.6%) compared with those not considering the specialty (61.1%). Students considering radiology were also more likely to perceive radiologists as providing direct surgical treatment (53.9% vs. 38.9%) and as performing surgical or interventional procedures (62.7% vs. 43.4%) compared with those not considering radiology Table 7.

**Table 7:** Associations between perceptions of radiologist roles and consideration of radiology.

Variable	Category	Not considering radiology(n = 198), n (%)	Considering radiology(n = 102), n (%)	p-value
Awareness of the difference between a radiologist and a radiographer	No	37 (18.7)	21 (20.6)	0.041
Awareness of the difference between a radiologist and a radiographer	Yes	121 (61.1)	72 (70.6)	
Awareness of the difference between a radiologist and a radiographer	Not sure	40 (20.2)	9 (8.8)	
Radiologists provide direct surgical treatment	No	121 (61.1)	47 (46.1)	0.013
Radiologists provide direct surgical treatment	Yes	77 (38.9)	55 (53.9)	
Radiologists perform surgical procedures	No	112 (56.6)	38 (37.3)	0.002
Radiologists perform surgical procedures	Yes	86 (43.4)	64 (62.7)	

### Radiology career preferences and associated reasons among medical students

Medical students’ reasons for preferring radiology as a future specialty included minimal patient contact (54.6%), a broad and varied knowledge base (47.3%), and competitive training (33.7%). Concerns related to radiation exposure (33.3%), an appealing physics or technology component (31.6%), and strong interest in the specialty (31.6%) were also commonly reported. Conversely, among students not selecting radiology, the most frequently reported reasons were greater interest in another specialty (70.3%), lack of interest in performing the same tasks continuously (69.1%), and concerns regarding radiation exposure (63.2%). Additional deterrents included minimal patient contact (46.6%), a perceived lack of variety within the specialty (42.9%), competitive training (41.9%), excessive patient interaction (28.1%), and knowing a radiologist (26.0%).

Regarding the preference between diagnostic and interventional radiology, a greater proportion of students expressed interest in interventional radiology (30.7%) compared with diagnostic radiology (17.3%), while 17.3% were equally interested in both; however, 34.7% reported being unsure Table 8.

**Table 8:** Radiology career consideration and reported reasons for and against choosing radiology.

Variable	Category	n	%
Considering radiology as a future specialty	Yes	102	23.9
Considering radiology as a future specialty	No	198	46.4
Considering radiology as a future specialty	Not sure	127	29.7
Likelihood of choosing interventional radiology after completing radiology residency	Very unlikely	64	15.0
Likelihood of choosing interventional radiology after completing radiology residency	Unlikely	80	18.7
Likelihood of choosing interventional radiology after completing radiology residency	Not sure	202	47.3
Likelihood of choosing interventional radiology after completing radiology residency	Likely	60	14.1
Likelihood of choosing interventional radiology after completing radiology residency	Very likely	21	4.9
Interest between diagnostic and interventional radiology	More interested in interventional radiology	131	30.7
Interest between diagnostic and interventional radiology	More interested in diagnostic radiology	74	17.3
Interest between diagnostic and interventional radiology	Equally interested	74	17.3
Interest between diagnostic and interventional radiology	Unsure	148	34.7
Reasons for selecting radiology as a specialty:	Minimal patient contact	233	54.6
Reasons for selecting radiology as a specialty:	Broad and extensive knowledge	202	47.3
Reasons for selecting radiology as a specialty:	Competitive training	144	33.7
Reasons for selecting radiology as a specialty:	Risk of radiation exposure	142	33.3
Reasons for selecting radiology as a specialty:	Physics/technology component is appealing	135	31.6
Reasons for selecting radiology as a specialty:	Very interested in the specialty	135	31.6
Reasons for selecting radiology as a specialty:	I know a radiologist	122	28.6
Reasons for selecting radiology as a specialty:	Too much patient interaction	90	21.1
Reasons for not selecting radiology as a specialty:	Interested in another specialty	300	70.3
Reasons for not selecting radiology as a specialty:	Interesting, but do not want to do it all the time	295	69.1
Reasons for not selecting radiology as a specialty:	Risk of radiation exposure	270	63.2
Reasons for not selecting radiology as a specialty:	Minimal patient contact	199	46.6
Reasons for not selecting radiology as a specialty:	Physics/technology component is not appealing	183	42.9
Reasons for not selecting radiology as a specialty:	Not varied enough	183	42.9
Reasons for not selecting radiology as a specialty:	Competitive training	179	41.9
Reasons for not selecting radiology as a specialty:	Too much patient interaction	120	28.1
Reasons for not selecting radiology as a specialty:	I know a radiologist	111	26.0

### Association between considering radiology as a future specialty and attitudinal factors

Students’ intention to consider radiology as a future specialty was significantly associated with multiple attitudinal and interpersonal factors. A markedly higher proportion of students considering radiology reported strong interest in the specialty and perceived radiology as offering extensive knowledge and competitive training opportunities compared with those not considering radiology (all *p* < 0.001). Awareness of radiation risk was also significantly associated with specialty choice (*p* = 0.014).

Preferences related to patient interaction showed significant associations. Students considering radiology were more likely to report that excessive patient interaction influenced their career considerations (*p* = 0.001), while preference for minimal patient contact was also significantly associated with considering radiology (*p* = 0.015). In addition, personal exposure to the field through knowing a radiologist was significantly associated with considering radiology as a future specialty (*p* < 0.001) Table 9.

**Table 9:** Associations between reported reasons for considering radiology and radiology career consideration.

Variable	Category	Not considering radiology(n = 198), n (%)	Considering radiology(n = 102), n (%)	p-value reported in manuscript
Very interested in the specialty	No	176 (88.9)	19 (18.6)	< 0.001
Very interested in the specialty	Yes	22 (11.1)	83 (81.4)	
Minimal patient contact	No	128 (64.6)	15 (14.7)	0.015
Minimal patient contact	Yes	70 (35.4)	87 (85.3)	
Too much patient interaction	No	165 (83.3)	70 (68.6)	0.001
Too much patient interaction	Yes	33 (16.7)	32 (31.4)	
Radiation risk	No	137 (69.2)	56 (54.9)	0.014
Radiation risk	Yes	61 (30.8)	46 (45.1)	
Competitive training	No	151 (76.3)	47 (46.1)	< 0.001
Competitive training	Yes	47 (23.7)	55 (53.9)	
Extensive knowledge	No	147 (74.2)	20 (19.6)	< 0.001
Extensive knowledge	Yes	51 (25.8)	82 (80.4)	
Knowing a radiologist	No	155 (78.3)	53 (52.0)	< 0.001
Knowing a radiologist	Yes	43 (21.7)	49 (48.0)	

The most influential factors in choosing radiology as a future specialty were lifestyle (57.8%), impact on patient care (52.2%), work environment (50.1%), intellectual challenges (43.8%), degree of patient contact (42.4%), procedures (41.5%), and job market (40.5%). The least important factors were participation in recruitment events (26.2%), participation in live didactic lectures (27.4%), and involvement in a professional radiology organization (28.3%) Table 10.

**Table 10:** Reported importance of factors in choosing radiology.

Factor	Reported percentage
Lifestyle	57.8
Impact on patient care	52.2
Work environment	50.1
Intellectual challenges	43.8
Degree of patient contact	42.4
Procedures	41.5
Job market	40.5
Experience during radiology rotations, clerkships, or electives	39.6
Experience of radiology during non-radiology rotations, clerkships, or electives	38.6
Advanced technology	37.7
Compensation	37.0
Role Models	34.4
Shadowing a radiologist	32.1
Radiology residents	31.4
Physics	30.0
Being involved in radiology research	30.0
Online curricula	29.7
Radiology interest group	29.3
Being involved in professional radiology organizations	28.3
Participation in live didactic lectures	27.4
Participation in recruitment events	26.2

Several perceived career-related factors were significantly associated with students’ intention to consider radiology as a future specialty. Lifestyle showed a strong association, with students who rated lifestyle as “very important” being more likely to consider radiology (p = 0.001). Compensation was also significantly associated with specialty consideration (p = 0.002), as were perceptions regarding the impact of radiology on patient care (p = 0.012). Educational and experiential factors played an important role. The perceived importance of radiological experience was significantly associated with considering radiology (p = 0.002), as was the importance placed on procedural involvement (p = 0.021). Students who valued technological knowledge were more likely to consider radiology (p = 0.037), highlighting the specialty’s technical appeal.

Workplace-related perceptions, including work environment (p = 0.020) and interaction with residents (p = 0.002), were also significantly associated with radiology preference. Additionally, the perceived importance of research involvement showed a significant relationship with considering radiology as a future specialty (p = 0.031). Collectively, these findings indicate that lifestyle, financial considerations, educational exposure, and professional environment are key determinants influencing medical students’ interest in radiology Table 11.

**Table 11:** Associations between ratings of radiology career factors and consideration of radiology.

Factor	χ² (df)	p-value
Impact on patient care	10.87 (3)	0.012
Compensation	14.63 (3)	0.002
Lifestyle	15.84 (3)	0.001
Experience during radiology placements	14.93 (3)	0.002
Procedures	9.71 (3)	0.021
Experience of radiology during other placements	8.37 (3)	0.039
Advanced technology	8.49 (3)	0.037
Work environment	9.83 (3)	0.020
Radiology residents	15.00 (3)	0.002
Involvement in radiology research	8.85 (3)	0.031

### AI, technology & future of radiology

Regarding perceptions of artificial intelligence (AI) in radiology, the majority of participants agreed that AI would enhance radiologists’ capabilities and improve efficiency (60.0%). However, nearly one-third of respondents agreed that AI would replace radiologists within their lifetime (32.1%). In addition, 40.0% of participants believed that the impact of AI alone would reduce the number of radiologists needed in the future. Despite these concerns, more than half of the respondents agreed that radiologists should embrace artificial intelligence and collaborate with the information technology industry to facilitate its implementation in clinical practice (54.8%).

When asked whether an introduction to tele-radiology would influence their choice of radiology as a future specialty, 34.0% of participants reported that it would influence their decision, whereas 23.7% indicated that it would not. A substantial proportion of respondents (42.4%) were uncertain about the potential influence of tele-radiology on their specialty choice.

Perceptions regarding the influence of tele-radiology were significantly associated with students’ intention to consider radiology as a future specialty (*p* < 0.001). A substantially higher proportion of students considering radiology reported that an introduction to tele-radiology would influence their career choice compared with those not considering radiology (56.9% vs. 24.2%). Conversely, students not considering radiology were more likely to report that tele-radiology would not influence their choice (34.8% vs. 14.7%) or that they were uncertain about its influence (40.9% vs. 28.4%) Table 12.

**Table 12:** Artificial intelligence attitudes and perceived teleradiology influence by radiology career consideration.

Item and displayed response	Not considering radiology(n = 198), n (%)	Considering radiology(n = 102), n (%)	p-value
Artificial intelligence will enhance radiologists’ capabilities and efficiency	120 (60.6)	54 (52.9)	0.059
AI will replace radiologists within the respondent’s lifetime	72 (36.4)	23 (22.5)	0.034
Artificial intelligence will reduce the number of radiologists needed	90 (45.5)	29 (28.4)	0.075
Radiologists should embrace artificial intelligence and collaborate with industry for its implementation	120 (60.6)	44 (43.1)	0.043
Would an introduction to teleradiology influence specialty choice? — Yes	48 (24.2)	58 (56.9)	<0.001

### Cognitive & personal influences on radiology interest

Regarding cognitive and perceptual skills relevant to radiology, confidence in visual–spatial skills was significantly associated with considering radiology as a future specialty, with students reporting confidence in these skills demonstrating higher odds of interest in radiology (p = 0.005). In contrast, although a majority of respondents believed that visual–spatial skills are important for a radiology career (63.0%), this perception was not significantly associated with specialty choice (p = 0.234). Enjoyment of diagnostic problem-solving tasks was reported by 63.0% of participants; however, this factor did not reach statistical significance in relation to considering radiology as a future specialty (p = 0.081). Notably, students who reported that diagnostic problem-solving increased the appeal of radiology were significantly more likely to consider radiology as a future specialty (p < 0.001) Table 13.

**Table 13:** Cognitive factors and their associations with consideration of radiology.

Cognitive factor	All participants(N = 427), n (%)	Association p-value(n = 300)
Confidence in visual–spatial skills	171 (40.0)	0.005
Belief that visual–spatial skills are important for radiology	269 (63.0)	0.234
Enjoyment of diagnostic problem-solving tasks	269 (63.0)	0.081
Diagnostic problem-solving increases appeal of radiology	169 (39.6)	< 0.001

### Regression

Multivariable logistic regression analysis was performed to identify factors independently associated with considering radiology as a future specialty. Of the 300 participants with valid outcome data, 267 (89.0%) had complete covariate data and were included in the multivariable model using a complete-case approach. Approximately 11% of eligible cases were excluded due to missing covariate data. No imputation techniques were applied. Variables with a p-value ≤ 0.20 in bivariate analyses, along with variables deemed clinically relevant, were entered into the multivariable logistic regression model.

The overall model was statistically significant (Omnibus χ^2^(11) = 44.13, p < 0.001) and demonstrated acceptable explanatory power (Cox & Snell R^2^ = 0.152; Nagelkerke R^2^ = 0.211). Model calibration was adequate, as indicated by a non-significant Hosmer–Lemeshow goodness-of-fit test (χ^2^(8) = 5.12, p = 0.745). Using a classification cut-off of 0.50, the model correctly classified 71.2% of cases, with high specificity (91.0%) and modest sensitivity (32.2%).

After adjustment for potential confounders, **exposure to radiology-related research** and **exposure to preclinical radiology lectures** remained independently associated with considering radiology as a future specialty. Students without exposure to radiology research had significantly lower odds of considering radiology compared with those who had research exposure (OR = 0.14, 95% CI 0.06–0.34, p < 0.001). Similarly, students without exposure to preclinical radiology lectures had lower odds of considering radiology compared with those exposed (OR = 0.26, 95% CI 0.12–0.54, p < 0.001).

Year of study was not significant overall (p = 0.289); however, one academic year category demonstrated significantly higher odds of considering radiology compared with the reference group (OR = 4.11, 95% CI 1.05–16.01, p = 0.042). Additionally, students who reported enjoying diagnostic problem-solving tasks were significantly more likely to consider radiology as a future specialty (OR = 3.35, 95% CI 1.39–8.09, p = 0.007).

In contrast, self-perceived visual–spatial ability and beliefs regarding the importance of visual–spatial skills were not independently associated with specialty choice in the adjusted model. Likewise, GPA, perceived work–life balance, and mentor or role-model influence were not statistically significant predictors (all p > 0.05).

Notably, **exposure to tele-radiology as an introduction to the field** was independently associated with increased odds of considering radiology as a future specialty (OR = 3.96, 95% CI 1.72–9.12, p = 0.001) Table 14.

**Table 14:** Multivariable logistic regression of factors associated with consideration of radiology.

Variable or contrast	Adjusted OR	95% CI	p-value
No exposure to radiology research	0.14	0.06–0.34	< 0.001
No exposure to preclinical radiology lectures	0.26	0.12–0.54	< 0.001
Year of study	4.11	1.05–16.01	0.042
Year of study — overall test	—	—	0.289
Enjoyment of diagnostic problem-solving tasks	3.35	1.39–8.09	0.007
Perceived influence of teleradiology	3.96	1.72–9.12	0.001

## Discussion

Radiology has a main role in medical practice, playing a critical role in diagnosis, disease monitoring, and interventional management across almost all clinical specialties. Despite the rapid expansion of its clinical and technological aspects, recruitment into radiology remains challenging in many regions, particularly in low- and middle-income settings. In Palestine, uncertainty concerning training pathways, insufficient undergraduate exposure, and understanding the radiologist’s role may influence career decision-making. Our study provides an insight into Palestinian medical students’ knowledge, perception, and intention regarding radiology as a future specialty, offering an evidence-based reference for understanding factors that may affect specialty choice.

Work–life balance, income, and diversity within the field were the most frequently reported determinants of specialty preference. These findings are broadly consistent with previous studies in Ireland and Saudi Arabia, which identified work environment, exposure, and lifestyle factors as key influences.

In the present study, lifestyle, perceived impact on patient care, and work environment were the strongest radiology-specific motivators [10,12,14]. Unlike some prior reports, financial incentives were not independently associated with specialty consideration, suggesting that stability and professional fulfillment may outweigh income expectations in this context [12,14].

We found the introduction of tele-radiology could positively influence the student’s intention to consider radiology as their future specialty, given the newly expanding practices, including remote work services and technological advancements that have been introduced to radiology [15]. Future studies should evaluate whether early exposure to tele-radiology influences long-term specialty choice.

No significant gender differences were observed, contrasting with some international findings suggesting a higher male preference for radiology. The year of study was also not independently associated with specialty choice, highlighting potential contextual differences in exposure and career development pathways [12,14].

A literature review by Levaillant et al. (2020) mentioned the regionally variant most influential factors in terms of specialty choice for medical students, which are work-life balance, their interest in the field, and academic background [16]. Students’ engagement, work–life balance, commitment to patient care, and mentorship have been reported as important factors influencing specialty choice [17]. These outside research results emphasize the crucial role of personal preferences in specialty selection.

Interest in another specialty was the main reason for not choosing radiology. While the previous literature found that concerns about radiation exposure are a discouraging factor [10,12], our results are consistent with those of Lynch et al. (2022), who found that these concerns influence the students’ choice of radiology, which was attributed to their greater awareness and interest in radiation protection rather than discouraging them. This disagreement calls for more studies to understand students’ perceptions and the motivations behind these preferences.

The misunderstanding of artificial intelligence (AI) concepts may mislead students’ influence on specialty choice [18]; therefore, many studies have called for more research to explore how AI could affect the specialty choice of medical students [14,18]. Emerging evidence from human-centered AI research suggests that generative AI in radiology is perceived as both an educational opportunity and a source of professional uncertainty. Such dual perceptions may shape specialty attractiveness among medical students, particularly in contexts where exposure to AI literacy is limited [15]. These concerns appeared to reflect fears of replacement rather than recognition of AI as a supportive clinical tool, consistent with mixed findings in previous studies [12]. These findings highlight the need for educational initiatives that accurately present the evolving role of artificial intelligence as a complementary rather than replacement technology.

Our study aims to assess the factors that influence Palestinian medical students’ interest in radiology. Early exposure, perceptions of work-life balance, radiologist role awareness, and engagement in diagnostic problem-solving activities were associated with greater interest in the field; however, family expectations and concerns related to medico-legal issues appeared to discourage students.

In interpreting these findings, it is evident that both educational experiences and personal perceptions play important roles in shaping students’ attitudes toward radiology. Based on these results, targeted educational and mentoring strategies -such as well –structured radiology curricula, diverse intellectual opportunities, addressing radiation and litigation concerns, and clarifying the radiologist’s role are recommended to support and encourage greater student engagement in radiology.

### Limitations and strengths of the study

#### Limitations.

This study has several limitations. First, the use of an online, voluntary survey distributed through social media platforms may have introduced selection bias, as participation depended on students’ digital accessibility, engagement with social media, and willingness to respond. Second, the reliance on self-reported data may have resulted in recall bias and social desirability bias, potentially affecting the accuracy of participants’ responses. Third, although the study included medical students from multiple Palestinian universities and achieved an adequate sample size, the use of convenience sampling may limit the generalizability of the findings to all Palestinian medical students and to medical students in other countries. Fourth, while multivariable regression was performed to adjust for measured confounders, the possibility of residual confounding from unmeasured factors cannot be excluded. Finally, the cross-sectional study design precludes establishing causal relationships, and no longitudinal follow-up was conducted to determine whether students’ stated career preferences ultimately translated into actual specialty choice. The adapted questionnaire and additional exploratory items did not undergo further psychometric validation, which may affect the interpretation of these measures.

#### Strength.

This study has several strengths. It included participants from multiple accredited medical schools across Palestine and represented students from different academic years, improving the diversity of the sample. The study employed a previously validated questionnaire and incorporated multivariable regression analysis to identify independent factors associated with specialty preference. Furthermore, it is among the first multicenter studies to examine determinants of interest in diagnostic radiology among Palestinian medical students.

## Conclusion

Palestinian medical students’ interest in diagnostic radiology was associated with educational exposure, cognitive engagement with diagnostic problem-solving, and perceptions of work–life balance, whereas demographic characteristics were not significantly associated with specialty preference. Early academic exposure to radiology, including preclinical teaching, research involvement, and elective experiences, emerged as one of the principal modifiable factor associated with greater consideration of radiology as a future career. Conversely, concerns regarding medico-legal issues, radiation exposure, and misconceptions about the role of radiologists were identified as important barriers. These findings may help inform the development of undergraduate radiology curricula, structured clinical exposure, and mentorship initiatives aimed at improving awareness, addressing misconceptions, and supporting informed career decision-making among medical students. Further multicenter and longitudinal studies are warranted to evaluate whether these educational strategies translate into increased recruitment into radiology training programs.

## Supporting information

S1 Appendix Study questionnaire. English-language questionnaire administered to assess factors associated with considering diagnostic radiology as a future specialty among Palestinian medical students.
(DOCX)

S1 Dataset Anonymized participant-level study data and codebook. Dataset underlying the reported findings, with definitions of variables, response coding, missing values, and transformations applied for participant privacy.
(XLSX)

S1 Checklist STROBE checklist. Completed STROBE checklist for the multicenter cross-sectional study.
(DOCX)

## References

[1] McManus IC, Livingston G, Katona C. The attractions of medicine: the generic motivations of medical school applicants in relation to demography, personality and achievement. BMC Med Educ. 2006;6:11. doi: 10.1186/1472-6920-6-11 16504048 PMC1397825

[2] DeZee KJ, Maurer D, Colt R, Shimeall W, Mallory R, Powers J, et al. Effect of financial remuneration on specialty choice of fourth-year U.S. medical students. Acad Med. 2011;86(2):187–93. doi: 10.1097/ACM.0b013e3182045ec9 21169785

[3] Al-Fouzan R, Al-Ajlan S, Marwan Y, Al-Saleh M. Factors affecting future specialty choice among medical students in Kuwait. Med Educ Online. 2012;17(1):19587. doi: 10.3402/meo.v17i0.19587 23272649 PMC3529360

[4] Ciechanowski PS, Russo JE, Katon WJ, Walker EA. Attachment theory in health care: the influence of relationship style on medical students’ specialty choice. Med Educ. 2004;38(3):262–70. doi: 10.1046/j.1365-2923.2004.01767.x 14996335

[5] Petrides KV, McManus IC. Mapping medical careers: questionnaire assessment of career preferences in medical school applicants and final-year students. BMC Med Educ. 2004;4:18. doi: 10.1186/1472-6920-4-18 15461786 PMC524180

[6] Dorsey ER, Jarjoura D, Rutecki GW. Influence of controllable lifestyle on recent trends in specialty choice by US medical students. JAMA. 2003;290(9):1173–8. doi: 10.1001/jama.290.9.1173 12952999

[7] Alsubaie N, Aldhofaian HS, Alhuwaimel L, Ruxshan N, Alghamdi F, Shamia A, et al. Specialty preferences and the factors influencing them among pre-clerkship medical students: the first study from Alfaisal University-College of Medicine, Saudi Arabia. Cureus. 2016;8(11):e894. doi: 10.7759/cureus.894 28018764 PMC5179192

[8] Health Service Executive (HSE), National Doctors Training and Planning (NDTP) Unit. Medical workforce report 2020–2021. Dublin: Health Service Executive; 2021. https://iitos.com/wp-content/uploads/2021/05/NDTP-Workforce-Report-2020-21.pdf

[9] HSE National Doctors Training and Planning (NDTP) Unit. Review of the Clinical Radiology Medical Workforce in Ireland. 2017. https://www.hse.ie/eng/staff/leadership-education-development/met/plan/specialty-specific-reviews/clinical-radiology-chapter-for-web-2017.pdf

[10] Udam NG, Obaje PA, Emegoakor AC, Edache EL, Obaje EO, Oladosu AO, et al. Factors influencing the choice of radiology as a specialty among clinical-year medical students in Nigeria: a multi-center cross-sectional study. BMC Med Educ. 2025;25(1):1452. doi: 10.1186/s12909-025-08067-w 41121301 PMC12539099

[11] European Society of Radiology (ESR). What the radiologist should know about artificial intelligence - an ESR white paper. Insights Imaging. 2019;10(1):44. doi: 10.1186/s13244-019-0738-2 30949865 PMC6449411

[12] Almuhanna AF, Alsultan DH, Almohsen DS, AlHuraish DS, AlRatrout FN, Alzanadi RH, et al. Factors influencing choosing diagnostic radiology as a specialty among medical students. Acad Radiol. 2025;32(4):2371–7. doi: 10.1016/j.acra.2024.12.015 39800602

[13] Alchallah MO, Ismail H, Dia T, Shibani M, Alzabibi MA, Mohsen F, et al. Assessing diagnostic radiology knowledge among Syrian medical undergraduates. Insights Imaging. 2020;11(1):124. doi: 10.1186/s13244-020-00937-9 33226458 PMC7683655

[14] Lynch T, Bockhold S, McNulty JP. Factors influencing the choice of radiology as a medical specialty in Ireland. Eur J Radiol. 2022;151:110297. doi: 10.1016/j.ejrad.2022.110297 35436759

[15] Morrison K, Mathur A, Bradshaw A, Wartmann T, Lundi S, Zandifar A, et al. A human-centered approach to identifying promises, risks, & challenges of text-to-image Generative AI in radiology. AIES. 2025;8(2):1758–70. doi: 10.1609/aies.v8i2.36672

[16] Levaillant M, Levaillant L, Lerolle N, Vallet B, Hamel-Broza J-F. Factors influencing medical students’ choice of specialization: a gender based systematic review. EClinicalMedicine. 2020;28:100589. doi: 10.1016/j.eclinm.2020.100589 33134904 PMC7588859

[17] Yang Y, Li J, Wu X, Wang J, Li W, Zhu Y, et al. Factors influencing subspecialty choice among medical students: a systematic review and meta-analysis. BMJ Open. 2019;9(3):e022097. doi: 10.1136/bmjopen-2018-022097 30850399 PMC6429728

[18] Neittaanmäki N. Swedish medical students’ attitudes toward artificial intelligence and effects on career plans: a survey. Front Educ. 2025;10:1517116. doi: 10.3389/feduc.2025.1517116

