# Supplementary material for: Factors associated with choosing diagnostic radiology as a future specialty among Palestinian medical students: a multicenter cross-sectional study
Source: PLoS One. 2026 Oct 29;21(10):e0361927. doi: 10.1371/journal.pone.0361927 (PMC13718955; doi:10.1371/journal.pone.0361927)
Supplement: Supplementary file 1 [file pone.0361927.s001.docx]

# Appendix I: Study Tool, The Questionnaire

**Section 1: Consent**

This questionnaire aims to explore the factors influencing medical students’ choice of diagnostic radiology as a future specialty. Your responses are confidential and will be used for academic purposes only. Participation is voluntary, and you may withdraw at any time before submitting the survey. All data will be anonymized.

**Do you consent to participate in this study?**

- Yes, I agree
- No, I do not agree

**Section 2: Demographics**

**Age:** _______

**Gender:**

- Male
- Female

**Marital Status:**

- Single
- Married
- Divorced
- Widowed

**Monthly Income:**

- < 2,500 NIS
- 2,500–5,000 NIS
- 5,000 NIS
- Other: _______

**Primary Source of Income:**

- Family support
- Scholarship
- Part-time work
- Other: _______

**Year of Study:**

- 2nd
- 3rd
- 4th
- 5th
- 6th
- Intern

**University:**

- Arab American University
- An-Najah National University
- Al-Quds University
- Palestine Polytechnic University
- Hebron University
- Washington University
- Al-Azhar University – Gaza
- Islamic University of Gaza (IUG)
- Other: _______

Your GPA. (If your university uses a percentile system, please indicate "not applicable.")

- <2.5
- 2.5-2.99
- >3
- Not applicable

**Please indicate your GPA; if your university uses a system out of 4, write "not applicable. If not.**

- Excellent (90–100%)
- Very Good (80–89.9%)
- Good (70–79.9%)
- Fair (60–69.9%)
- Not applicable

**Section 3: Current Specialty Interests**

**Broadly, which category best describes your interest?**

- Medicine
- Surgery
- Both

**Are you currently interested in a particular specialty?**

- Yes
- No
- Maybe

**Which specialty are you most interested in? (Select one)**

- Anatomical Pathology
- Anesthesia
- Basic Science
- Cardiac Surgery
- Critical Care Medicine
- Diagnostic Radiology
- Dermatology
- Emergency Medicine
- Family Medicine
- Forensic Medicine
- General Practice
- General Surgery
- Internal Medicine
- Neurology
- Neurosurgery
- Obstetrics and Gynecology
- Occupational Medicine
- Ophthalmology
- Orthopedics
- Otorhinolaryngology (ENT)
- Pediatrics
- Pediatric Surgery
- Physical Medicine and Rehabilitation
- Psychiatry
- Radiation Oncology
- Urology
- Other: _______

**Factors Influencing Specialty Choice**

To what extent do the following factors affect your decision when choosing your specialty?

(Options: No influence / Minor influence / Moderate influence / Strong influence)

- Academic examination results
- Competition for training programs
- Diversity within the field
- Degree of patient contact
- Family expectations
- Income potential or future earnings
- Interest in acute patient management
- Current exposure or knowledge of the field
- Length of training
- Work–life balance
- Mentor or role model influence
- Potential litigation or medico-legal issues
- Social status of the specialty
- Working environment and professional relationships

**Section 4: Exposure to Radiology**

**To date, have you had any exposure to radiology?**

- Yes
- No

If yes, what type of exposure have you had? (Select all that apply)

- Preclinical radiology lectures
- Radiology elective/rotation
- Radiology research
- Radiology conferences
- Radiology interest group activities
- Other: _______

**Have you completed a formal radiology elective or clerkship?**

- Yes
- No

If yes, why did you choose to take radiology-related experience?

- I am considering radiology as a specialty
- To expand my clinical exposure
- I am not considering radiology but recognize its importance
- Other: _______

If yes, did your experience turn you toward or away from radiology?

- Toward
- Away
- Neither

**Do you think the amount of exposure to radiology during medical school (especially in clinical years) is:**

- Too little
- Just right
- Too much
- I am not yet in those clinical years

**Section 5: Perception and Awareness of the Radiologist’s Role**

**Radiologists play an important role in diagnosis and patient management.**

- Agree
- Disagree
- Neutral

**Are you aware of the difference between a radiologist and a radiographer?**

- Yes
- No
- Not sure

**In your opinion, do radiologists perform the following roles? (Yes / No)**

- Diagnose patients
- Direct medical treatment
- Direct surgical treatment
- Meet with patients
- Perform surgical procedures
- Perform conventional x-ray/radiographic examinations (e.g., chest and musculoskeletal x-ray)
- Direct radiographers to optimize/set the parameters used for different imaging modalities
- Have Responsibility for the Practical radiation projection of patients undergoing x-ray or CT examination

**Section 6 of 8**

Radiology Choice with Associated Reasons for Preference among Medical Student

Are you considering radiology as a future specialty?

- Yes
- No
- Not sure

Are the following reasons why you are considering radiology? (Yes / No)

- Very interested in the specialty
- Physics/technology component is appealing
- Minimal patient contact
- Too much patient interaction
- Risk of radiation
- Competitive training
- Very varied/extensive knowledge
- I know a radiologist

If there are any other factors, please specify.

Are the following reasons why you are not considering radiology? (Yes / No)

- Interesting but do not want to do it all the time
- Physics/technology component not appealing
- Minimal patient contact
- Too much patient interaction
- Risk of radiation exposure
- My mind is made up to pursue another specialty
- Competitive training
- Not varied enough
- I know a radiologist

If there are any other factors, please specify.

Please rate how important each of the following was in your decision to choose radiology as your specialty.

not important / somewhat important / important / very important

- Degree of patient contact
- Intellectual challenge
- Impact on patient care
- Role models (mentors, faculty advisor, dean, family member)
- Compensation
- Lifestyle
- Experience on Radiology rotation/clerkship/elective/selective
- Procedures
- Experience with Radiology during a non-Radiology rotation / clerkship /elective / selective
- Job market
- Physics
- Advanced/cutting edge technology
- Work environment
- Shadowing a radiologist
- Online curricula (such as self-directed computer-based models or prerecorded didactic lectures)
- Radiology interest group
- Radiology residents
- Being involved in radiology research
- Participating at a recruitment event (e.g., residency fair)
- Live didactic lectures
- Being involved in professional Radiology organizations

For each of the following statements, indicate whether you strongly agree, agree, are neutral, disagree, or strongly disagree.

strongly disagree / disagree / neither disagree nor agree / agree / strongly agree

- Radiologists have patient contact
- Radiologists are exposed to a significant degree of radiation over the course of their careers
- Radiologists play a vital role in diagnosis and patient management.
- Radiologists have a “controllable” lifestyle (i.e., relatively predictable work hours).
- Radiologists’ compensation (salary) is greater than many other physician specialties.
- New techniques/technologies are frequently being developed in the field of Radiology.
- Radiology is monotonous.
- In the future, most radiology studies will be interpreted from off-site locations (i.e., national or international).
- Radiology positions are becoming more limited in the current job market

How likely is it that you will choose interventional radiology as a career after completing Radiology residency

- Very unlikely
- Unlikely
- Not sure
- Likely
- Very likely

Between diagnostic radiology and interventional radiology, you are?

- More interested in interventional radiology
- More interested in diagnostic radiology
- Equally interested
- Unsure

**Section 7 of 8**

AI, Technology & Future of Radiology

Artificial intelligence will enhance radiologists' capabilities and make them more efficient.

- Strongly disagree
- Disagree
- Somewhat disagree
- Neither agree nor disagree
- Somewhat agree
- Agree
- Strongly agree

Artificial intelligence will replace radiologists in my lifetime.

- Strongly disagree
- Disagree
- Somewhat disagree
- Neither agree nor disagree
- Somewhat agree
- Agree
- Strongly agree

The impact of artificial intelligence alone will reduce the number of radiologists that are needed.

- Strongly disagree
- Disagree
- Somewhat disagree
- Neither agree nor disagree
- Somewhat agree
- Agree
- Strongly agree

Radiologists should embrace artificial intelligence, and work with IT industry for its application

- Strongly disagree
- Disagree
- Somewhat disagree
- Neither agree nor disagree
- Somewhat agree
- Agree
- Strongly agree

Would teleradiology—an introduction to the field of radiology—influences your choice?

- Yes
- No
- Not Sure

**Section 8 of 8**

Cognitive & Personal Influences on Radiology Interest

How confident are you in your visual–spatial skills (e.g., mentally rotating anatomical structures, visualizing 3D relationships)?

- Very confident
- confident
- Neutral
- Not confident
- Very unconfident

Do you believe strong visual-spatial abilities are important for a career in radiology?

- Yes
- No
- Unsure

Do you enjoy tasks that involve diagnostic problem-solving (such as puzzles, pattern recognition, or analytic reasoning)?

- Yes
- No
- Neutral

Does this enjoyment make radiology more appealing to you as a specialty?

- Yes
- No
- Neutral
