# Supplementary material for: Factors associated with choosing diagnostic radiology as a future specialty among Palestinian medical students: a multicenter cross-sectional study
Source: PLoS One. 2026 Oct 29;21(10):e0361927. doi: 10.1371/journal.pone.0361927 (PMC13718955; doi:10.1371/journal.pone.0361927)
Supplement: Supplementary file 2 [file pone.0361927.s003.docx]

STROBE Statement—checklist of items that should be included in reports of observational studies

|  | Item No. | Recommendation | Page  No. | Relevant text from manuscript |
| --- | --- | --- | --- | --- |
| **Title and abstract** | 1 | (*a*) Indicate the study’s design with a commonly used term in the title or the abstract | 1-2 | A multi-institutional cross-sectional study” |
|  |  | (*b*) Provide in the abstract an informative and balanced summary of what was done and what was found | 2 | Structured abstract (Background, Methods, Results, Conclusions) |
| Introduction | | | |  |
| Background/rationale | 2 | Explain the scientific background and rationale for the investigation being reported | 3-4 | Introduction paragraphs describing global radiology recruitment and regional context |
| Objectives | 3 | State specific objectives, including any prespecified hypotheses | 4 | “This study aimed to identify factors associated with…” |
| Methods | | | |  |
| Study design | 4 | Present key elements of study design early in the paper | 4 | A multicenter cross-sectional study was conducted from 2 November 2025 to 5 February 2026 |
| Setting | 5 | Describe the setting, locations, and relevant dates, including periods of recruitment, exposure, follow-up, and data collection | 4 | Palestinian medical schools in the West Bank and Gaza Strip; data collection from 2 November 2025 to 5 February 2026; recruitment through social media groups and student representatives |
| Participants | 6 | (*a*) *Cohort study*—Give the eligibility criteria, and the sources and methods of selection of participants. Describe methods of follow-up  *Case-control study*—Give the eligibility criteria, and the sources and methods of case ascertainment and control selection. Give the rationale for the choice of cases and controls  *Cross-sectional study*—Give the eligibility criteria, and the sources and methods of selection of participants | 4 | Refer to Study population, including eligibility, voluntary consent, and exclusion criteria. |
|  |  | (*b*) *Cohort study*—For matched studies, give matching criteria and number of exposed and unexposed  *Case-control study*—For matched studies, give matching criteria and the number of controls per case |  | Not applicable — cross-sectional study without matching. |
| Variables | 7 | Clearly define all outcomes, exposures, predictors, potential confounders, and effect modifiers. Give diagnostic criteria, if applicable | 5 | Definition of outcome variable (considering radiology) and predictor variables |
| Data sources/ measurement | 8* | For each variable of interest, give sources of data and details of methods of assessment (measurement). Describe comparability of assessment methods if there is more than one group | *4-5* | *English-language questionnaire adapted from Almuhanna et al. [12], with additional exploratory items reviewed by two consultant radiologists; no additional pilot testing or psychometric validation. Questionnaire provided as S1 Appendix.* |
| Bias | 9 | Describe any efforts to address potential sources of bias | 4,12 | Refer to eligibility criteria, questionnaire review, and the Limitations section. Do not claim that selection or recall bias was eliminate |
| Study size | 10 | Explain how the study size was arrived at | 4 | Sample size calculation description |

Continued on next page

| Quantitative variables | 11 | Explain how quantitative variables were handled in the analyses. If applicable, describe which groupings were chosen and why | 5 | Age is summarized using the mean, standard deviation, and range. Monthly income and GPA are presented as categories in Table 1. Categorical variables are summarized using frequencies and percentages. The rationale for grouping or recoding variables is not fully described. |
| --- | --- | --- | --- | --- |
| Statistical methods | 12 | (*a*) Describe all statistical methods, including those used to control for confounding | 5-6 | Bivariate associations were assessed using Pearson’s chi-square test or Fisher’s exact test, as appropriate. Variables with p ≤ 0.20 in bivariate analysis, together with clinically relevant factors, were entered into multivariable binary logistic regression. Odds ratios and 95% confidence intervals were reported. Model fit was assessed using the Omnibus test, Hosmer–Lemeshow test, and pseudo-R² statistics. |
|  |  | (*b*) Describe any methods used to examine subgroups and interactions | Not applicable | No subgroup-specific or interaction analyses are reported in the manuscript |
|  |  | (*c*) Explain how missing data were addressed | 5-6 | Complete-case analysis was used. Participants with incomplete responses for variables included in the multivariable model were excluded from the adjusted analysis. No imputation was performed. |
|  |  | (*d*) *Cohort study*—If applicable, explain how loss to follow-up was addressed  *Case-control study*—If applicable, explain how matching of cases and controls was addressed  *Cross-sectional study*—If applicable, describe analytical methods taking account of sampling strategy | 5-6 | Participants were recruited using non-probability convenience sampling through broad dissemination of the questionnaire. No academic-year quotas were specified. Sampling weights or other sampling-design adjustments are not described. |
|  |  | (*e*) Describe any sensitivity analyses | Not applicable | No sensitivity analyses are reported in the manuscript. |
| Results | | | | |
| Participants | 13* | (a) Report numbers of individuals at each stage of study—eg numbers potentially eligible, examined for eligibility, confirmed eligible, included in the study, completing follow-up, and analysed | 6 | The manuscript reports that 436 students accessed the survey, 427 completed the questionnaire, 300 were included in the primary outcome analysis, and 267 were included in multivariable regression. |
|  |  | (b) Give reasons for non-participation at each stage | 6 | Nine individuals who accessed the survey did not complete the questionnaire. Of the 427 completed responses, 127 were not included in the primary outcome analysis; the reason is not explicitly described in the manuscript. A further 33 participants were excluded from multivariable regression because of incomplete covariate data. |
|  |  | (c) Consider use of a flow diagram | 4 | Figure 1 summarizes participant recruitment, inclusion, and the final analytical samples. |
| Descriptive data | 14* | (a) Give characteristics of study participants (eg demographic, clinical, social) and information on exposures and potential confounders | 6-8 | Table 1 presents demographic, socioeconomic, and academic characteristics. Subsequent Results sections and tables describe specialty interests, radiology exposure, perceptions, and other study variables. |
|  |  | (b) Indicate number of participants with missing data for each variable of interest | 6 | The manuscript reports 33 exclusions from multivariable regression because of incomplete covariate data. Variable-specific missing-data counts and the distinction between missing responses and uncertain outcome responses are not fully reported. |
|  |  | (c) *Cohort study*—Summarise follow-up time (eg, average and total amount) | Not applicable | Not applicable — cross-sectional study with no follow-up period. |
| Outcome data | 15* | *Cohort study*—Report numbers of outcome events or summary measures over time | *6-9* | *Not applicable. This was a cross-sectional study.* |
|  |  | *Case-control study—*Report numbers in each exposure category, or summary measures of exposure | *Not applicable* | *Not applicable. This was a cross-sectional study.* |
|  |  | *Cross-sectional study—*Report numbers of outcome events or summary measures | *6-9* | *“Among 300 valid responses, 102 students (34.0%) reported considering radiology as a future specialty.”* |
| Main results | 16 | (*a*) Give unadjusted estimates and, if applicable, confounder-adjusted estimates and their precision (eg, 95% confidence interval). Make clear which confounders were adjusted for and why they were included | 9-10 | Bivariate associations are presented in the Results tables. Adjusted odds ratios, 95% confidence intervals, and p-values are presented in Table 14. Statistical analysis describes the criteria for selecting variables for the multivariable model. |
|  |  | (*b*) Report category boundaries when continuous variables were categorized | Not applicable | Table 1 reports monthly income categories of <2,500 NIS, 2,500–5,000 NIS, and >5,000 NIS, together with GPA categories. The derivation of the reported GPA categories is not fully explained. |
|  |  | (*c*) If relevant, consider translating estimates of relative risk into absolute risk for a meaningful time | Not applicable | Not applicable. The study reports cross-sectional associations rather than risk estimates over a defined follow-up period. |

Continued on next page

| Other analyses | 17 | Report other analyses done—eg analyses of subgroups and interactions, and sensitivity analyses | 7-9 | Bivariate analyses examining associations between consideration of radiology and specialty-choice factors, educational exposure, perceptions, and cognitive factors are reported. No subgroup, interaction, or sensitivity analyses are reported. |
| --- | --- | --- | --- | --- |
| Discussion | | | | |
| Key results | 18 | Summarise key results with reference to study objectives | 10 | The Discussion summarizes associations between radiology interest, educational exposure, cognitive engagement, perceptions of work–life balance, and teleradiology, with reference to the study objectives. |
| Limitations | 19 | Discuss limitations of the study, taking into account sources of potential bias or imprecision. Discuss both direction and magnitude of any potential bias | 12 | Limitations include voluntary online recruitment, convenience sampling, selection bias, self-reported responses, recall and social desirability bias, residual confounding, limited generalizability, the cross-sectional design, and absence of further psychometric validation. The direction and magnitude of potential biases are not quantified. |
| Interpretation | 20 | Give a cautious overall interpretation of results considering objectives, limitations, multiplicity of analyses, results from similar studies, and other relevant evidence | 10-12 | The Discussion compares the findings with previous studies and considers their educational implications. The limitations of the cross-sectional design and the need for longitudinal research are acknowledged. |
| Generalisability | 21 | Discuss the generalisability (external validity) of the study results | 12 | The manuscript acknowledges that convenience sampling may limit generalizability to all Palestinian medical students and to medical students in other countries. |
| Other information | |  | | |
| Funding | 22 | Give the source of funding and the role of the funders for the present study and, if applicable, for the original study on which the present article is based | 15 | This research received no external funding.” |

*Give information separately for cases and controls in case-control studies and, if applicable, for exposed and unexposed groups in cohort and cross-sectional studies.

**Note:** An Explanation and Elaboration article discusses each checklist item and gives methodological background and published examples of transparent reporting. The STROBE checklist is best used in conjunction with this article (freely available on the Web sites of PLoS Medicine at http://www.plosmedicine.org/, Annals of Internal Medicine at http://www.annals.org/, and Epidemiology at http://www.epidem.com/). Information on the STROBE Initiative is available at www.strobe-statement.org.
