# Supplementary material for: Factors associated with choosing diagnostic radiology as a future specialty among Palestinian medical students: a multicenter cross-sectional study
Source: PLoS One. 2026 Oct 29;21(10):e0361927. doi: 10.1371/journal.pone.0361927 (PMC13718955; doi:10.1371/journal.pone.0361927)
Supplement: Supplementary file 4 [file pone.0361927.s006.docx]

Response to the editor and reviewers (PONE-D-26-36866)

Factors associated with choosing diagnostic radiology as a future specialty among Palestinian medical students: a multicenter cross-sectional study

Dear Dr. Faggioni,

We thank you and the reviewers for your assessment and constructive comments.

## 1 Manuscript style and presentation

**Comment:** Please ensure that the manuscript meets PLOS ONE style requirements, including file naming.

**Response:** We have added the previously missing Tables 1–14 with captions, clarified the Methods headings, made selected wording and punctuation corrections, and corrected the DOI presentation in reference 15. These are the presentation changes made in the current manuscript; we do not claim a comprehensive style or submission-system file-naming audit.

## 2 Data availability

**Comment:** Make all data underlying the findings freely available, or explain any justified restrictions.

**Response:** We replaced the previous restricted-access/upon-request statement. The revised manuscript states: “All de-identified data underlying the findings reported in this study, together with a codebook describing the variables, response coding, and missing values, are provided in S1 Dataset.” This response records the change to the manuscript statement. Upload of the actual dataset and codebook, and amendment of the online submission statement, are not established by the manuscript file.

## 3 Ethics statement

**Comment:** The ethics statement should appear only in Methods.

**Response:** We consolidated the ethics statement under “Ethical approval and informed consent” in Methods and removed its duplicate from Declarations. The statement identifies the Arab American University Institutional Review Board, approval number J-2025/A/67/N, electronic informed consent, voluntary participation, confidentiality and anonymity.

## 4 Missing tables

**Comment:** Please include Tables 1–14 referenced in the manuscript.

**Response:** We have inserted all 14 tables as editable tables, with captions, alongside the corresponding Results passages. The original submitted PDF referred to these tables but did not contain them. Their addition presents the tabulated results; it does not represent a new statistical analysis.

## 5 Supporting Information captions

**Comment:** Include Supporting Information captions at the end of the manuscript and update in-text citations.

**Response:** The Methods now cites S1 Appendix, and the data-availability statement identifies S1 Dataset. We also included the questionnaire text within the manuscript under “Appendix I: Study Tool, The Questionnaire.” However, a separate Supporting Information caption list has not been added at the end, and the Appendix I heading has not been harmonized with the S1 Appendix citation. This requirement is therefore only partly addressed in the current version.

# Reviewer 1

## 1 Study period

**Comment:** Please mention the study period.

**Response:** We explicitly added the study period, 2 November 2025 to 5 February 2026, to the opening Methods paragraph. These dates were already reported in the Abstract of the original submission.

## 2 Questionnaire source and modification

**Comment:** Please cite Almuhanna et al. (2025) and explain the questionnaire modification process.

**Response:** We added the in-text citation [12] to the source instrument in Methods; the bibliographic reference was already present in the original submission. We revised the instrument description, summarized its domains, and removed the statement that it was used without modification. The current text describes two additional cognitive and visual–spatial items, developed following a focused literature review, review by two consultant radiologists, and minor wording revisions. It also states that no additional pilot testing or psychometric validation was performed. We added this limitation to the Limitations section.

## 3 Questionnaire language

**Comment:** What was the language of the questionnaire used?

**Response:** We explicitly state that the questionnaire was administered in English. The questionnaire text is included in Appendix I, and Methods refers to it as S1 Appendix.

## 4 Licensed statistical software

**Comment:** Please mention details of the licensed SPSS software.

**Response:** We expanded the software description to identify IBM SPSS Statistics, version 27.0 (IBM Corp., Armonk, NY, USA), used under an institutional licence held by Arab American University, Palestine.

## 5 Recent studies in the Discussion

**Comment:** The Discussion could be strengthened by adding recent studies, including Almubarak et al. (2026) and Tahir et al. (2024).

**Response:** The two suggested studies have not been added to the current manuscript. Our Discussion changes are limited to correcting the incomplete sentence about factors influencing specialty choice and changing the citation for the human-centered generative-AI statement from [10] to [15]. The suggested literature additions are therefore not claimed as a completed revision.”.

# Reviewer 2

Comment summary: The reviewer provided a favorable assessment of the study and discussed workforce relevance, educational exposure, AI perceptions, selection bias, generalizability, interventional radiology interest and the absence of causal inference.

Response: We thank the reviewer for the detailed assessment and supportive comments. The existing limitations concerning voluntary online participation, convenience sampling, generalizability and the cross-sectional design have been retained. We added an explicit limitation concerning the absence of further psychometric validation of the adapted questionnaire. No new analysis or causal claim has been introduced in response to these comments.

# Other changes made

We corrected 247/427 from 56.7% to 57.8% in Current Specialty Interest, corrected the heading “fStudy variables” to “Study variables,” made minor wording and punctuation edits in the Introduction and the paragraph preceding Table 2, and standardized the DOI presentation in reference 15. The reference list retains its original 18 entries; no new reference was added.

Sincerely,
Dr. Mahdi Aljamal
Corresponding author, on behalf of the authors
