# Supplementary material for: Factors associated with choosing diagnostic radiology as a future specialty among Palestinian medical students: a multicenter cross-sectional study
Source: PLoS One. 2026 Oct 29;21(10):e0361927. doi: 10.1371/journal.pone.0361927 (PMC13718955; doi:10.1371/journal.pone.0361927)
Supplement: Supplementary file 5 [file pone.0361927.s004.docx]

Research Article

PONE-D-26-36866

**Factors Associated with Choosing Diagnostic Radiology as a Future**

Workforce planning and especially physician specialty choices are tracked poorly for some fields such as radiology. There is a severe global shortage of radiologists that has been further exacerbated by post covid increases in remote and part-time practice, upward market pressures on radiologist compensation and increased volumes of imaging studies in aging populations. Fears that AI will replace radiologists are rampant while integrated algorithms to help radiologists streamline work are not yet “ready for prime time”.

In this manuscript, the authors evaluated factors influencing medical students’ choice of radiology as a future subspecialty via a previously validated questionnaire over the November 2025 - February 2026 time interval. The sample size included 427 students at the Arab American University and outreach to all accredited medical schools in Palestine (West Bank and Gaza Strip; 56.7% women) which 102 students indicated considering radiology. Perhaps not surprisingly, lack of exposure to radiology research (OR = 0.14, 95% CI 0.06–0.34, p < 0.001) and lack of preclinical radiology teaching (OR = 0.26, 95% CI 0.12–0.54, p < 0.001) were independently associated with a lower likelihood of considering radiology. Trends in medical school curricula over the past decade + have decreased exposure to radiology as a career choice with a move to integrated curricula that include medical imaging more often as a tool rather than a profession in the medical decision making process.

Enjoyment of diagnostic problem-solving tasks (OR = 3.35, 95% CI 1.39–8.09, p = 0.007) and exposure to tele-radiology (OR = 3.96, 95% CI 1.72–9.12, p = 0.001) were independently associated with higher likelihood. Lifestyle, impact on patient care, and work environment were the most frequently reported motivating factors. Perceived impact of AI is a particularly important factor that was included in the surveys. Certainly impressions by faculty and/or other influential mentors that AI will *replace* radiologists is a relevant issue. Interestingly this factor did not arise as a driver of interest or lack of interest in entering radiology as a future career yet the majority of participants agreed that AI would enhance radiologists’ capabilities and improve efficiency (60.0%). Nearly one-third of respondents agreed that AI would replace radiologists within their lifetime (32.1%).

Students were surveyed in their 2^nd^ to 6^th^ year through voluntary informed consent. The authors conclude that engagement of undergraduate students in the role of radiology and appealing technological and lifestyle aspects of the field could increase student interest.

Importantly, the survey questions addressed a range of specialty selection so it is unlikely there is a selection bias re representation of interest in radiology relative to other fields. However, as the authors note, there could be a selection bis due to the multifactorial distribution plan using WhatsApp and other social media platforms. Awareness of the distinction between a radiologist and a radiographer was understandably more common among students considering radiology.

The mean age of the survey participants was 21.75 yrs, which is young compared to medical students in the US so results may differ in countries with variables such as medical student program design and cultural influences.

The authors found that interventional radiology is particularly appealing to medical students, reflecting the trend toward IR.

Importantly, the authors draw no causal relationship between the student.

The article is well written and offers important relevance.
