# Supplementary material for: Factors associated with choosing diagnostic radiology as a future specialty among Palestinian medical students: a multicenter cross-sectional study
Source: PLoS One. 2026 Oct 29;21(10):e0361927. doi: 10.1371/journal.pone.0361927 (PMC13718955; doi:10.1371/journal.pone.0361927)
Supplement: Peer review history

# Peer review: Decision Letter 0

**DOI:** 10.1371/journal.pone.0361927.r001

**Authors:** Lorenzo Faggioni

28 Sep 2026

Dear Dr. aljamal,

Thank you for submitting your manuscript to PLOS One. After careful consideration, we feel that it has merit but does not fully meet PLOS One’s publication criteria as it currently stands. Therefore, we invite you to submit a revised version of the manuscript that addresses the points raised during the review process.

Please submit your revised manuscript by Nov 27 2026 11:59PM. If you will need more time than this to complete your revisions, please reply to this message or contact the journal office at plosone@plos.org. When you're ready to submit your revision, log on to https://www.editorialmanager.com/pone/ and select the 'Submissions Needing Revision' folder to locate your manuscript file.

- A letter that responds to each point raised by the academic editor and reviewer(s). You should upload this letter as a separate file labeled 'Response to Reviewers'.
- A marked-up copy of your manuscript that highlights changes made to the original version. You should upload this as a separate file labeled 'Revised Manuscript with Track Changes'.
- An unmarked version of your revised paper without tracked changes. You should upload this as a separate file labeled 'Manuscript'.

If you would like to make changes to your financial disclosure, please include your updated statement in your cover letter. Guidelines for resubmitting your figure files are available below the reviewer comments at the end of this letter.

If applicable, we recommend that you deposit your laboratory protocols in protocols.io to enhance the reproducibility of your results. Protocols.io assigns your protocol its own identifier (DOI) so that it can be cited independently in the future. For instructions see: https://journals.plos.org/plosone/s/submission-guidelines#loc-laboratory-protocols. Additionally, PLOS One offers an option for publishing peer-reviewed Lab Protocol articles, which describe protocols hosted on protocols.io. Read more information on sharing protocols at https://plos.org/protocols?utm_medium=editorial-email&utm_source=authorletters&utm_campaign=protocols.

As the corresponding author, your ORCID iD is verified in the submission system and will appear in the published article. PLOS supports the use of ORCID, and we encourage all coauthors to register for an ORCID iD and use it as well. Please encourage your coauthors to verify their ORCID iD within the submission system before final acceptance, as unverified ORCID iDs will not appear in the published article. *Only*  the individual author can complete the verification step; PLOS staff *cannot*  verify ORCID iDs on behalf of authors.

We look forward to receiving your revised manuscript.

Kind regards,

Lorenzo Faggioni, M.D., Ph.D.

Academic Editor

PLOS One

Journal Requirements:

When submitting your revision, we need you to address these additional requirements.

1. Please ensure that your manuscript meets PLOS ONE's style requirements, including those for file naming. The PLOS ONE style templates can be found at https://journals.plos.org/plosone/s/file?id=wjVg/PLOSOne_formatting_sample_main_body.pdf and https://journals.plos.org/plosone/s/file?id=ba62/PLOSOne_formatting_sample_title_authors_affiliations.pdf

2. In the online submission form, you indicated that [The datasets generated and/or analyzed during the current study are available from the corresponding author upon reasonable request.].

All PLOS journals now require all data underlying the findings described in their manuscript to be freely available to other researchers, either 1. In a public repository, 2. Within the manuscript itself, or 3. Uploaded as supplementary information.

This policy applies to all data except where public deposition would breach compliance with the protocol approved by your research ethics board. If your data cannot be made publicly available for ethical or legal reasons (e.g., public availability would compromise patient privacy), please explain your reasons on resubmission and your exemption request will be escalated for approval.

3. Your ethics statement should only appear in the Methods section of your manuscript. If your ethics statement is written in any section besides the Methods, please move it to the Methods section and delete it from any other section. Please ensure that your ethics statement is included in your manuscript, as the ethics statement entered into the online submission form will not be published alongside your manuscript.

4. Please include a copy of Tables 1-14 which you refer to in your text on pages 7-12.

5. Please include captions for your Supporting Information files at the end of your manuscript, and update any in-text citations to match accordingly. Please see our Supporting Information guidelines for more information: http://journals.plos.org/plosone/s/supporting-information.

If the reviewer comments include a recommendation to cite specific previously published works, please review and evaluate these publications to determine whether they are relevant and should be cited. There is no requirement to cite these works unless the editor has indicated otherwise.

Please review your reference list to ensure that it is complete and correct. If you have cited papers that have been retracted, please include the rationale for doing so in the manuscript text, or remove these references and replace them with relevant current references. Any changes to the reference list should be mentioned in the rebuttal letter that accompanies your revised manuscript. If you need to cite a retracted article, indicate the article’s retracted status in the References list and also include a citation and full reference for the retraction notice.

[Note: HTML markup is below. Please do not edit.]

Reviewers' comments:

Reviewer's Responses to Questions

**Comments to the Author**

1. Is the manuscript technically sound, and do the data support the conclusions?

Reviewer #1: Yes

Reviewer #2: Yes

**********

2. Has the statistical analysis been performed appropriately and rigorously?

Reviewer #1: Yes

Reviewer #2: Yes

**********

3. Have the authors made all data underlying the findings in their manuscript fully available?

The PLOS Data policy

Reviewer #1: Yes

Reviewer #2: Yes

**********

4. Is the manuscript presented in an intelligible fashion and written in standard English?

Reviewer #1: Yes

Reviewer #2: Yes

**********

Reviewer #1: I read the study with great interest

Please mention the study period.

Please add a reference to the questionnaire by Almuhanna et al. (2025) used in your study, and explain the process of modifying your questionnaire in detail.

What was the language of the questionnaire used? Further details would be good

Statistical Package for the Social Sciences (SPSS) 27. Please mention details of the licensed software.

Discussion can be strengthened by adding recent studies for example:

Almubarak, H. A., Almaymoni, A. A. H. A. M., Alharethi, W. S. Z., Moalwi, O. H. M., Almathahibi, L. S. A., Alqahtani, R. S. A., Khuzayyim, A. A., Almoghamer, H. D. M., Badawi, F. A. H., Alshahrani, W. A. A., Daghriri, L. A. A., Almehery, K. A. A., Alsamghan, A., & Mahmood, S. E. (2026). Factors influencing medical students’ decisions on specialty training in radiology at King Khalid University, Abha, Saudi Arabia: A cross-sectional study. Medicine, 105(22), e49096. https://doi.org/10.1097/MD.0000000000049096

Tahir MJ, Jamil H, Jabbar R, et al. Radiology as a career among medical students of Pakistan: a cross-sectional study. Med (United States). Medicine (Baltim). 2024;103:e38156.

Reviewer #2: Research Article

PONE-D-26-36866

Factors Associated with Choosing Diagnostic Radiology as a Future

Workforce planning and especially physician specialty choices are tracked poorly for some fields such as radiology. There is a severe global shortage of radiologists that has been further exacerbated by post covid increases in remote and part-time practice, upward market pressures on radiologist compensation and increased volumes of imaging studies in aging populations. Fears that AI will replace radiologists are rampant while integrated algorithms to help radiologists streamline work are not yet “ready for prime time”.

In this manuscript, the authors evaluated factors influencing medical students’ choice of radiology as a future subspecialty via a previously validated questionnaire over the November 2025 - February 2026 time interval. The sample size included 427 students at the Arab American University and outreach to all accredited medical schools in Palestine (West Bank and Gaza Strip; 56.7% women) which 102 students indicated considering radiology. Perhaps not surprisingly, lack of exposure to radiology research (OR = 0.14, 95% CI 0.06–0.34, p < 0.001) and lack of preclinical radiology teaching (OR = 0.26, 95% CI 0.12–0.54, p < 0.001) were independently associated with a lower likelihood of considering radiology. Trends in medical school curricula over the past decade + have decreased exposure to radiology as a career choice with a move to integrated curricula that include medical imaging more often as a tool rather than a profession in the medical decision making process.

Enjoyment of diagnostic problem-solving tasks (OR = 3.35, 95% CI 1.39–8.09, p = 0.007) and exposure to tele-radiology (OR = 3.96, 95% CI 1.72–9.12, p = 0.001) were independently associated with higher likelihood. Lifestyle, impact on patient care, and work environment were the most frequently reported motivating factors. Perceived impact of AI is a particularly important factor that was included in the surveys. Certainly impressions by faculty and/or other influential mentors that AI will replace radiologists is a relevant issue. Interestingly this factor did not arise as a driver of interest or lack of interest in entering radiology as a future career yet the majority of participants agreed that AI would enhance radiologists’ capabilities and improve efficiency (60.0%). Nearly one-third of respondents agreed that AI would replace radiologists within their lifetime (32.1%).

Students were surveyed in their 2nd to 6th year through voluntary informed consent. The authors conclude that engagement of undergraduate students in the role of radiology and appealing technological and lifestyle aspects of the field could increase student interest.

Importantly, the survey questions addressed a range of specialty selection so it is unlikely there is a selection bias re representation of interest in radiology relative to other fields. However, as the authors note, there could be a selection bis due to the multifactorial distribution plan using WhatsApp and other social media platforms. Awareness of the distinction between a radiologist and a radiographer was understandably more common among students considering radiology.

The mean age of the survey participants was 21.75 yrs, which is young compared to medical students in the US so results may differ in countries with variables such as medical student program design and cultural influences.

The authors found that interventional radiology is particularly appealing to medical students, reflecting the trend toward IR.

Importantly, the authors draw no causal relationship between the student.

The article is well written and offers important relevance.

**********

what does this mean?). If published, this will include your full peer review and any attached files.

If you choose “no”, your identity will remain anonymous but your review may still be made public.

**Do you want your identity to be public for this peer review?**  For information about this choice, including consent withdrawal, please see our Privacy Policy

Reviewer #1: **Yes:**  Syed Esam Mahmood

Reviewer #2: No

**********

[NOTE: If reviewer comments were submitted as an attachment file, they will be attached to this email and accessible via the submission site. Please log into your account, locate the manuscript record, and check for the action link "View Attachments". If this link does not appear, there are no attachment files.]

To ensure your figures meet our technical requirements, please review our figure guidelines: https://journals.plos.org/plosone/s/figures

You may also use PLOS’s free figure tool, NAAS, to help you prepare publication quality figures: https://journals.plos.org/plosone/s/figures#loc-tools-for-figure-preparation.

NAAS will assess whether your figures meet our technical requirements by comparing each figure against our figure specifications.

Attachment Submitted filename: PLOS ONE_rad workforce review.docx

# Author comment: Author response to Decision Letter 1

**DOI:** 10.1371/journal.pone.0361927.r002

6 Oct 2026

Response to the editor and reviewers (PONE-D-26-36866)

Factors associated with choosing diagnostic radiology as a future specialty among Palestinian medical students: a multicenter cross-sectional study

Dear Dr. Faggioni,

We thank you and the reviewers for your assessment and constructive comments.

1 Manuscript style and presentation

Comment: Please ensure that the manuscript meets PLOS ONE style requirements, including file naming.

Response: We have added the previously missing Tables 1–14 with captions, clarified the Methods headings, made selected wording and punctuation corrections, and corrected the DOI presentation in reference 15. These are the presentation changes made in the current manuscript; we do not claim a comprehensive style or submission-system file-naming audit.

2 Data availability

Comment: Make all data underlying the findings freely available, or explain any justified restrictions.

Response: We replaced the previous restricted-access/upon-request statement. The revised manuscript states: “All de-identified data underlying the findings reported in this study, together with a codebook describing the variables, response coding, and missing values, are provided in S1 Dataset.” This response records the change to the manuscript statement. Upload of the actual dataset and codebook, and amendment of the online submission statement, are not established by the manuscript file.

3 Ethics statement

Comment: The ethics statement should appear only in Methods.

Response: We consolidated the ethics statement under “Ethical approval and informed consent” in Methods and removed its duplicate from Declarations. The statement identifies the Arab American University Institutional Review Board, approval number J-2025/A/67/N, electronic informed consent, voluntary participation, confidentiality and anonymity.

4 Missing tables

Comment: Please include Tables 1–14 referenced in the manuscript.

Response: We have inserted all 14 tables as editable tables, with captions, alongside the corresponding Results passages. The original submitted PDF referred to these tables but did not contain them. Their addition presents the tabulated results; it does not represent a new statistical analysis.

5 Supporting Information captions

Comment: Include Supporting Information captions at the end of the manuscript and update in-text citations.

Response: The Methods now cites S1 Appendix, and the data-availability statement identifies S1 Dataset. We also included the questionnaire text within the manuscript under “Appendix I: Study Tool, The Questionnaire.” However, a separate Supporting Information caption list has not been added at the end, and the Appendix I heading has not been harmonized with the S1 Appendix citation. This requirement is therefore only partly addressed in the current version.

Reviewer 1

1 Study period

Comment: Please mention the study period.

Response: We explicitly added the study period, 2 November 2025 to 5 February 2026, to the opening Methods paragraph. These dates were already reported in the Abstract of the original submission.

2 Questionnaire source and modification

Comment: Please cite Almuhanna et al. (2025) and explain the questionnaire modification process.

Response: We added the in-text citation [12] to the source instrument in Methods; the bibliographic reference was already present in the original submission. We revised the instrument description, summarized its domains, and removed the statement that it was used without modification. The current text describes two additional cognitive and visual–spatial items, developed following a focused literature review, review by two consultant radiologists, and minor wording revisions. It also states that no additional pilot testing or psychometric validation was performed. We added this limitation to the Limitations section.

3 Questionnaire language

Comment: What was the language of the questionnaire used?

Response: We explicitly state that the questionnaire was administered in English. The questionnaire text is included in Appendix I, and Methods refers to it as S1 Appendix.

4 Licensed statistical software

Comment: Please mention details of the licensed SPSS software.

Response: We expanded the software description to identify IBM SPSS Statistics, version 27.0 (IBM Corp., Armonk, NY, USA), used under an institutional licence held by Arab American University, Palestine.

5 Recent studies in the Discussion

Comment: The Discussion could be strengthened by adding recent studies, including Almubarak et al. (2026) and Tahir et al. (2024).

Response: The two suggested studies have not been added to the current manuscript. Our Discussion changes are limited to correcting the incomplete sentence about factors influencing specialty choice and changing the citation for the human-centered generative-AI statement from [10] to [15]. The suggested literature additions are therefore not claimed as a completed revision.”.

Reviewer 2

Comment summary: The reviewer provided a favorable assessment of the study and discussed workforce relevance, educational exposure, AI perceptions, selection bias, generalizability, interventional radiology interest and the absence of causal inference.

Response: We thank the reviewer for the detailed assessment and supportive comments. The existing limitations concerning voluntary online participation, convenience sampling, generalizability and the cross-sectional design have been retained. We added an explicit limitation concerning the absence of further psychometric validation of the adapted questionnaire. No new analysis or causal claim has been introduced in response to these comments.

Other changes made

We corrected 247/427 from 56.7% to 57.8% in Current Specialty Interest, corrected the heading “fStudy variables” to “Study variables,” made minor wording and punctuation edits in the Introduction and the paragraph preceding Table 2, and standardized the DOI presentation in reference 15. The reference list retains its original 18 entries; no new reference was added.

Sincerely,

Dr. Mahdi Aljamal

Corresponding author, on behalf of the authors

Attachment Submitted filename: PONE 36866 Response to reviewers.docx

# Peer review: Decision Letter 1

**DOI:** 10.1371/journal.pone.0361927.r003

**Authors:** Lorenzo Faggioni

19 Oct 2026

Factors Associated with Choosing Diagnostic Radiology as a Future Specialty among Palestinian Medical Students: A Multicenter Cross-Sectional Study

PONE-D-26-36866R1

Dear Dr. aljamal,

We’re pleased to inform you that your manuscript has been judged scientifically suitable for publication and will be formally accepted for publication once it meets all outstanding technical requirements.

Within one week, you’ll receive an e-mail detailing the required amendments. When these have been addressed, you’ll receive a formal acceptance letter and your manuscript will be scheduled for publication.

An invoice will be generated when your article is formally accepted. Please note, if your institution has a publishing partnership with PLOS and your article meets the relevant criteria, all or part of your publication costs will be covered. Please make sure your user information is up-to-date by logging into Editorial Manager at Editorial Manager® and clicking the ‘Update My Information' link at the top of the page. For questions related to billing, please contact billing support.

If your institution or institutions have a press office, please notify them about your upcoming paper to help maximize its impact. If they’ll be preparing press materials, please inform our press team as soon as possible -- no later than 48 hours after receiving the formal acceptance. Your manuscript will remain under strict press embargo until 2 pm Eastern Time on the date of publication. For more information, please contact onepress@plos.org.

Kind regards,

Lorenzo Faggioni, M.D., Ph.D.

Academic Editor

PLOS One

Reviewers' comments:

Reviewer's Responses to Questions

**Comments to the Author**

Reviewer #2: All comments have been addressed

**********

2. Is the manuscript technically sound, and do the data support the conclusions?

Reviewer #2: Yes

**********

3. Has the statistical analysis been performed appropriately and rigorously?

Reviewer #2: Yes

**********

4. Have the authors made all data underlying the findings in their manuscript fully available?

The PLOS Data policy

Reviewer #2: Yes

**********

5. Is the manuscript presented in an intelligible fashion and written in standard English?

Reviewer #2: Yes

**********

Reviewer #2: the authors have addressed the issues raised in the initial review and included previously missing tables for completeness

**********

what does this mean?). If published, this will include your full peer review and any attached files.

If you choose “no”, your identity will remain anonymous but your review may still be made public.

**Do you want your identity to be public for this peer review?**  For information about this choice, including consent withdrawal, please see our Privacy Policy

Reviewer #2: No

**********

# Editor report: Acceptance letter

**DOI:** 10.1371/journal.pone.0361927.r004

**Authors:** Lorenzo Faggioni

PONE-D-26-36866R1

PLOS One

Dear Dr. aljamal,

I'm pleased to inform you that your manuscript has been deemed suitable for publication in PLOS One. Congratulations! Your manuscript is now being handed over to our production team.

At this stage, our production department will prepare your paper for publication. This includes ensuring the following:

* All references, tables, and figures are properly cited

* All relevant supporting information is included in the manuscript submission,

* There are no issues that prevent the paper from being properly typeset

You will receive further instructions from the production team, including instructions on how to review your proof when it is ready. Please keep in mind that we are working through a large volume of accepted articles, so please give us a few days to review your paper and let you know the next and final steps.

Lastly, if your institution or institutions have a press office, please let them know about your upcoming paper now to help maximize its impact. If they'll be preparing press materials, please inform our press team within the next 48 hours. Your manuscript will remain under strict press embargo until 2 pm Eastern Time on the date of publication. For more information, please contact onepress@plos.org.

You will receive an invoice from PLOS for your publication fee after your manuscript has reached the completed accept phase. If you receive an email requesting payment before acceptance or for any other service, this may be a phishing scheme. Learn how to identify phishing emails and protect your accounts at https://explore.plos.org/phishing.

If we can help with anything else, please email us at customercare@plos.org.

Thank you for submitting your work to PLOS One and supporting open access.

Kind regards,

PLOS One Editorial Office Staff

on behalf of

Dr. Lorenzo Faggioni

Academic Editor

PLOS One
